# Trends in the management of anophthalmic sockets and external ocular prostheses among oculoplastic surgeons: a web-based study

**DOI:** 10.5935/0004-2749.2025-0006

**Published:** 2025-09-10

**Authors:** Alicia Galindo-Ferreiro, Elvira Martinez-Fernandez, Carolina Pereira Bigheti, Denise Cassia Moreira Zornoff, Hortensia Sanchez-Tocino, Silvana Artioli Schellini

**Affiliations:** 1 Department of Ophthalmology, Rio Hortega University Hospital, Valladolid, Spain; 2 Department of Ophthalmology, Faculdade de Medicina, Universidade Estadual Paulista “Júlio de Mesquita Filho”, Botucatu, SP, Brazil; 3 Distance Education and Health Information Technology Center, Faculdade de Medicina, Universidade Estadual Paulista “Júlio de Mesquita Filho”, Botucatu, SP, Brazil

**Keywords:** Anophthalmos, Eye, artificial, Polymethyl methacrylate, Polyethylene, Surgeons, Surveys and questionnaires, Brazil, Spain

## Abstract

**Purpose:**

This study aimed to evaluate the practices employed by oculoplastic surgeons in the assessment and management of anophthalmic sockets and external ocular prostheses.

**Methods:**

Oculoplastic surgeons from two countries, who specialized in the management of anophthalmic sockets, participated in a web-based survey. Data collected included demographics, types of surgery, implant use, external ocular prostheses management (including fabrication and cleaning), complications encountered, and follow-up times. The frequencies and distributions of the responses were statistically analyzed.

**Results:**

A total of 177 oculoplastic surgeons participated, 113 (63.8%) from Brazil, the remainder from Spain. Evisceration was the preferred surgical procedure of 149 (84.2%) surgeons. The most commonly reported indication for enucleation was a painful blind eye (n=103, 58.1%; both Brazil and Spain, p<0.001). Brazilian surgeons preferred polymethyl methacrylate implants (n=65, 57.5%), while Spanish surgeons favored porous polyethylene implants (n=53, 82.8%; p<0.001). Discharge was the most frequently observed clinical feature during socket evaluation (n=164, 92.6%; p<0.001). Brazilian surgeons recommended daily (n=53, 46.9%) or weekly (n=41, 36.2%) cleaning of exter nal ocular prostheses, while Spanish surgeons more commonly recommended monthly cleaning (n=31, 48.4%; p<0.001). The majority of Brazilian surgeons (n=83, 73.4%) advised patients to remove their external ocular prostheses at night. Only a small number of Spanish surgeons (n=3, 4.6%) suggested this practice (p<0.001). Overall, the follow-up recommendations varied, with 70 (39.5%) surgeons recommending follow-up based on indivi dual case needs, and 59 (33.3%) suggesting annual visits (p<0.001). The primary indications for external ocular prostheses replacement were edge damage (n=75, 42.3%) and loss of volume (n=68, 38.4%). The replacement intervals given typically ranged from 1 to 5 years (n=92, 51.9%; p<0.001).

**Conclusion:**

Oculoplastic surgeons in Brazil and Spain demonstrated similar practices in the management of anophthalmic sockets. However, notable differences were observed in the choice of implant materials, cleaning protocols, and recommendations regarding external ocular prostheses removal during sleep.

## INTRODUCTION

An anophthalmic socket is a well-defined clinical condition. Combining orbital implants with external ocular prostheses (EOP) in patients with this condition is a well-established means of improving aesthetic outcomes^(1)^. However, there is a notable gap in the literature regarding the ongoing management of these patients. Anophthalmic patients are typically managed by oculoplastic surgeons in collaboration with ophthalmologists, and EOP cleaning is generally performed by the patient under their supervision^(1^,^2)^.

Care providers are tasked with providing clear and precise instructions on the appropriate care of both the prosthesis and conditions of the socket associated with anophthalmia. However, there is currently no consensus or standardized protocol regarding these care practices, particularly concerning the cleaning and handling of EOPS, and follow-up visits^(2^,^3)^. Additionally, communication between healthcare professionals and patients is often suboptimal^(3)^.

This study aims to evaluate professional practices in the routine examination, care, and management of anophthalmic sockets with EOP, as conducted by oculoplastic surgeons in Brazil and Spain. The study explores the underlying causes of anophthalmia and the preferred surgical techniques and orbital implants among oculoplastic surgeons. The findings may serve as a valuable resource, enhancing the management of patients with anophthalmic sockets.

## METHODS

This qualitative, web-based study was conducted between September and December 2021. Participants were oculoplastic surgeons with expertise in anophthalmic socket management, practicing in Brazil or Spain. We aimed to evaluate the profile of patients with ano ph thalmic sockets, and the treatment approaches and management practices among the participants when dealing with anophthalmic sockets and EOPs. The study adhered to the principles of the 1964 Declaration of Helsinki and its later amendments. It received ethical approval from the Ethics Committee of UNESP–Botucatu Medical School (approval no. 18.618-970) and the Ethics Committee of the Rio Hortega University Hospital (approval no. PI259-20).

Participants were identified through professional medical societies, social media platforms, and general web searches. They were invited to participate via emails sent to their institutional addresses. The invitation detailed the purpose of the study and provided a link to an electronic questionnaire available in Portuguese^(4)^ or Spanish^(5)^. A follow-up email was sent two weeks later to improve response rates.

The questionnaire was developed by the authors and comprised 27 questions, open-ended, dichotomous and multiple-choice questions in the official language of each country. It was designed using adaptive logic, enabling the display of follow-up questions or comment boxes based on previous responses. REDCap (Research Electronic Data Capture) and Google Forms^©^ (Google LLC, Alphabet Inc., 2021) were utilized in the design and dissemination of the survey.

All primary questions were mandatory. Some required a single response, while others allowed multiple selections, yielding a variable number of total responses. The questionnaire gathered information on the respondents’ demographics, their clinical experience with anophthalmic sockets, surgical preferences, use of orbital implants, postoperative outcomes, EOP management practices, observed complications in anophthalmic sockets, complications associated with EOPs during follow-up, and follow-up frequency.

The study responses were returned anonymously. However, the country of origin of each participant was recorded to enable comparative analysis between Brazilian and Spanish respondents. To ensure respondent anonymity, a unique numerical identifier was automatically assigned to each internet-connected device at the time of data submission.

### Statistical analysis

Data were compiled in Microsoft Excel (Microsoft Corp., Redmond, WA, USA) and analyzed using frequency distributions and percentages. Non-parametric statistical tests and the chi-square test were applied to assess statistical significance (p value <0.05).

## RESULTS

The questionnaire was distributed to 709 ophthalmologists–379 in Brazil and 330 in Spain. A total of 177 (25%) oculoplastic specialists responded, of whom 113 (63.8%) were from Brazil. The majority of respondents (n=140, 79%) had completed their medical training after the year 2000 and reported a high level of experience in the management of anophthalmic sockets.

Evisceration was the most commonly performed surgical technique (n=149, 84.2%). Respondents from Brazil reported limited availability of orbital implants and prostheses, with 63 (55.8%) indicating that such resources were not provided through the public healthcare system, and 52 (46.4%) stating that they were not covered by private health insurance. In contrast, all Spanish participants reported that implants and prostheses were publicly provided.

### Clinical and surgical approaches

The most frequently cited indication for eye removal was a painful blind eye (n=103, 58.1%). This was a statistically significant finding in both Brazil (p<0.001) and Spain (p=0.003). The next most common reasons were phthisis bulbi (n=49, 27.6%) and ocular trauma (n=20, 11.2%). The frequencies of all causes are shown in table 1.

**Table 1:** Reasons for enucleation and evisceration among Brazilian and Spanish oculoplastic surgeons and the implants used

Reason for enucleation/evisceration	Brazil (n=113)	Spain (n=64)	Brazil + Spain (n=177)
Painful blind eye	68 (60.1%) *	35 (54.6%) *	103 (58.1%) *
Phthisis bulbi	32 (28.3%)	17 (26.5%)	49 (27.6%) *
Ocular trauma	19 (16.8%)	1 (1.5%)	20 (11.2%)
Anophthalmic socket with previous surgery	13 (11.5%)	6 (9.3%)	19 (10.7%)
Perforated eye	9 (7.9%)	0	9 (5%)
Tumor	3 (2.6%)	3 (4.6%)	6 (3.3%)
Complicated endophthalmitis	1 (0.8%)	0	1(0.5%)
Chi-square	(6) 153.88	(2) 16.10	(7) 323.74
p-value	<0.001	<0.001	<0.001

Implant	Brazil (Total=113)	Spain (Total=64)	Brazil + Spain (Total=177)
Porous polyethylene	27 (23.8%)	53 (82.8%) *	80 (45.1%) *
Polymethyl methacrylate	65 (57.5%) *	3 (4.6%)	68 (38.4%) *
Synthetic hydroxyapatite	8 (7%)	6 (0.9%)	14 (7.9%)
Glass	7 (6.1%)	0	7 (3.9%)
Other	4 (3.5%)	1 (1.5%)	5 (2.8%)
No implant	2 (1.7%)	1 (1.5%)	3 (1.6%)
Chi-square	(4) 115.32	(2) 70.53	(4) 143.14

* Statistically significant values.

Spherical orbital implants were the most commonly utilized, as reported by 88.4% of Brazilian and 89% of Spanish surgeons. Brazilian respondents showed a preference for polymethyl methacrylate (PMMA) implants (n=65, 57.5%), whereas Spanish specialists favored porous polyethylene (PP) implants (n=53, 82.8%). This was a statistically significant difference (p<0.001) (Tables 1 and 2).

**Table 2:** Orbital implants used by Brazilian and Spanish oculoplastic surgeons and complications observed in patients with anophthalmic sockets

Implant	Brazil (n=113)	Spain (n=64)	Brazil + Spain (N=177)
Porous polyethylene	27 (23.8%)	53 (82.8%) *	80 (45.1%) *
Polymethyl methacrylate	65 (57.5%) *	3 (4.6%)	68 (38.4%) *
Synthetic hydroxyapatite	8 (7%)	6 (0.9%)	14 (7.9%)
Glass	7 (6.1%)	0	7 (3.9%)
Other	4 (3.5%)	1 (1.5%)	5 (2.8%)
No implant	2 (1.7%)	1 (1.5%)	3 (1.6%)
Chi-square	(4) 115.32	(2) 70.53	(4) 143.14

Anophthalmic socket complications	Brazil (n=113)	Spain (n=64)	Brazil + Spain (N=177)
Discharge	97 (85.8%) *	67 (98.5%) *	164 (92.6%) *
Fornix shortening	59 (52.2%) *	34 (53.1%)	93 (52.5%) *
Lack of volume	29 (25.6%)	26 (40.6%)	55 (31.1%)
Conjunctival papillae	26 (23%)	28 (43.7%)	54 (30.5%)
Pyogenic granulomas	32 (28.3)	11 (17.1%)	43 (24.2%)
Scleral, conjunctival-tenon dehiscence	31 (27.4%)	11 (17.1%)	42 (23.7%)
Implant extrusion	16 (14.1%)	14 (21.8%)	30 (16.9%)
Prostheses mobility	10 (8.8%)	15 (23.4%)	25 (14.1%)
Chi-square	(8) 117.31	(7) 82.61	(8) 216.63

Grade of anophthalmic socket contraction	Brazil (n=113)	Spain (n=64)	Brazil + Spain (N=177)
Mild, with minimum fornix shortening	79 (69.9%) *	49 (76.5%) *	128 (72.3%) *
Intense, with severe fornix shortening	22 (19.4%)	2 (3.1%)	24 (13.5%)
No contraction	11 (9.7%)	13 (20.3%)	24 (13.5%)
Chi-square	(2) 71.38	(1) 37.76	(2) 122.91

* Statistically significant values. p-value <0.001.

Discharge around the prosthetic eye was the most frequently observed complication in anophthalmic socket evaluations (n=164, 92.6%). This was followed by deep superior sulcus deformity (n=125, 70.6%), ptosis (n=95, 53.6%), fornix shortening (n=93, 52.5%), volume deficiency (n=55, 31.1%), conjunctival papillae (n=54, 30.5%), pyogenic granulomas (n=43, 24.2%), wound dehiscence (n=42, 23.7%), and implant extrusion (n=30, 16.9%). Mild socket contraction was frequently reported in both countries, referred to by 79 ophthalmologists in Brazil (69.9%) and 49 in Spain (76.6%) (p<0.001). However, severe contraction with pronounced fornix shortening was more frequently observed in Brazil (n=22, 19.4%) (Table 2). Common complications with the EOP itself included surface deposits (n=70, 39.5%), scratches (n=62, 35%), and poor hygiene (n=54, 30.5%), finding statistically significant differences between the two groups (p<0.001) (Table 2).

### External ocular prosthesis management

EOP management practices varied between the two countries. In Brazil, the majority of surgeons recommended either daily (n=53, 46.9%) or weekly (n=41, 36.2%) cleaning of the prosthesis. However, most Spanish surgeons advised monthly cleaning (n=31, 48.4%), a difference that was statistically significant (p<0.001) (Table 3). Additionally, a significant proportion of Brazilian surgeons (n=83, 73.4%) advised patients to remove the prosthesis before sleeping, whereas only a small minority of Spanish surgeons (n=3, 4.6%) recommended this practice (p<0.001) (Table 3).

**Table 3:** Practices of Brazilian and Spanish oculoplastic surgeons with anophthalmic patients with external ocular prostheses

Recommended EOP cleaning frequency	Brazil (n=113)	Spain (n=64)	Brazil + Spain (N=177)
Once a week	41 (36.2%) *	18 (28.1%)	59 (33.3%) *
Daily	53 (46.9%) *	5 (7.8%)	58 (32.7%) *
Once a month	9 (7.9%)	31(48.4%) *	40 (22.5%)
Other	8 (7.0%)	10 (15.5%)	18 (10.0%)
Chi-square	(4) 95.98	(2) 6.78	(5) 116.05
p-value	<0.0001	<0.0001	<0.0001

Recommend removing EOP to sleep	83 (73.4%) *	3 (4.6%)	32 (18%)
Chi-square	(1) 24.86	(1) 52.56	(1) 0.14
p-value	<0.0001	<0.0001	=0.7070

Annual reassessment frequency	Brazil (n=113)	Spain (n=64)	Brazil + Spain (N=177)
Depends on the case	40 (35.3%)	30 (46.8%)	70 (39.5%) *
Once a year	34 (30%)	25 (39%)	59 (33.3%)
Twice a year	35 (30.9%)	7 (10.9%)	42 (23.7%)
Chi-square	(3) 30.50	(2) 11.28	(3) 55.14
p-value	<0.0001	=0.0036	<0.0001

Parameters for EOP replacement	*p*-value	Spain (n=64)	Brazil + Spain (N=177)
Edge damage	39 (34.5%) *	36 (56.2%)	75 (42.3%) *
Lack of volume	28 (24.7%)	40 (6.2%)	68 (38.4%)
Scratches	7 (6.1%)	33 (51.5%)	40 (22.5%)
Mobility	14 (12.3%)	17 (26.5%)	31 (17.5%)
Deposits	12 (10.6%)	18 (28.1%)	30 (16.9%)
Chi-square	(6) 61.63	(5) 35.36	(6) 107.79
p-value	<0.001	<0.001	<0.001

Prostheses replacement time	Brazil (n=113)	Spain (n=64)	Brazil + Spain (N=177)
1–5 years	59 (52.2%) *	33 (51.5%)	92 (51.9%) *
5–10 years	44 (38.9%)	26 (40.6%)	70 (39.5%)
Chi-square	(2) 35.27	(2) 19.91	(2) 55.14
p-value	<0.001	<0.001	<0.001

EOP= external ocular prostheses; -=alternative not available in that country.

* Statistically significant values.

Follow-up frequencies reported for patients with EOPs varied. While 70 respondents (39.5%) indicated that reassessment intervals depended on the indivi dual case, 59 (33.3%) recommended annual follow-ups (p<0.001) (Table 3).

The primary criteria for recommending EOP replacement included edge damage (75; 42.3%), loss of volume (68; 38.4%), and the presence of scratches (40; 22.5%). The most commonly reported replacement interval was every 1–5 years (92; 51.9%) (p<0.001) (Table 3).

## DISCUSSION

This study examined the management of anophthalmic sockets by oculoplastic surgeons from two continents, revealing diverse approaches to clinical care. Given the limited literature on this subject and the absence of standardized treatment protocols, our findings contribute valuable insights into this underexplored topic^(1^,^2)^.

A greater number of responses were obtained from Brazil, likely reflecting the greater number of practicing oculoplastic surgeons there. The overall response rate was 25%, lower than that obtained in previous studies (33.7%^(3)^; 46%^(6)^; 58%^(7)^), yet adequate for a reasonable sample size.

The majority of respondents had graduated within the last two decades, suggesting that they possessed substantial clinical experience in managing anophthalmic sockets and EOPs. Consistent with the findings of a previous study^(8)^, the most commonly reported indication for eye removal was a blind, painful eye. This is the terminal manifestation of various ocular pathologies and a leading cause of acquired anophthalmia^(9)^.

Among all of the respondents, evisceration was the preferred surgical technique over enucleation. This is in line with the current global preference for evisceration in non-tumoral cases due to its lesser surgical trauma and superior aesthetic outcomes^(8^,^10)^.

Orbital implant material preferences among oculoplastic surgeons have evolved over time in parallel with technological advances. In the United States, hydroxyapatite was the standard in the early 1990s^(11)^. However, by 2002, PP had become the material of choice^(12)^. Similarly, UK surgeons surveyed in 2004 were found to predominantly use PP^(13)^. In the present study, Brazilian surgeons preferred polymethyl methacrylate (PMMA) implants, corroborating the results of a national survey conducted in 2012^(14)^. This preference may be driven by cost considerations, as PMMA is more affordable and orbital implants are not reimbursed by the Brazilian public health system. In contrast, surgeons in Spain, where implants are funded by the public health service, predominantly used PP implants. This was also consistent with previous findings^(15)^. Most respondents favored spherical orbital implants, which are the most widely used implant shape internationally^(14^,^16)^.

As documented in previous studies^(17^-^20)^, our respondents reported a high incidence of discharge in anophthalmic sockets. While discharge may signal conjunctival inflammation, it can be exacerbated by scratches or deposits on the surface of the EOP or inadequate hygiene^(21)^. Additional contributing factors can include tear film abnormalities, impaired lacrimal drainage, and eyelid dysfunction.

Clinical signs such as deep superior sulcus, ptosis, and socket contraction were prevalent, even after adequate rehabilitation. These often reflect orbital volume deficiency^(22)^. Despite optimal implant sizing, factors such as the positioning, shape, and volume of the EOP can affect the contour of the superior sulcus^(12^,^23)^. Deep superior sulcus and ptosis are also characteristic of anophthalmic socket syndrome^(24)^, which results from the posterior displacement of orbital tissue following enucleation or evisceration^(25)^. Even with careful implant placement, the levator palpebrae superioris and superior rectus muscles may shift toward the orbital apex, creating a volume void in the superior orbit^(25)^.

Ptosis can also arise from repeated eyelid manipulation during EOP insertion and removal. This can lead to levator aponeurosis dehiscence, a phenomenon analogous to ptosis caused by long-term contact lens use, chronic eye rubbing, and floppy eyelid syndrome^(26^,^27)^. Rather than tissue elongation, altered positioning appears to be the underlying cause. In predisposed individuals, prolon ged EOP wear may contribute to this phenomenon. Adjustments in EOP design can improve eyelid height, but this can sometimes induce lagophthalmos^(27)^.

Progressive fornix shortening is a recognized sequela of long-term prosthetic use^(28)^. It is likely due to repeated mechanical trauma to the conjunctiva. Mild fornix shortening was most frequently reported by our respondents, over severe fornix shortening and no contraction. Successful rehabilitation requires adequate fornix depth to ensure prosthesis retention and satisfactory cosmetic outcomes. Inadequate depth can significantly affect a patient’s appearance and psychosocial well-being^(29)^.

EOP maintenance practices varied widely among surgeons. Consistent with prior local data^(2)^, daily cleaning and nighttime removal were most commonly recommended in Brazil. In contrast, most Spanish surgeons advised monthly cleaning and continuous wear, consistent with other international reports^(30)^. Higher blinking frequency has been linked to increased discharge and prosthesis removal, prompting Spanish clinicians to advise against daily removal^(19^,^23)^.

In both countries, follow-up frequency was typically individualized. Chronic EOP use, particularly with older prostheses, is associated with discharge, surface scratching, and poor hygiene, which can lead to biofilm formation and chronic inflammation. These conditions can, in turn, cause giant papillary conjunctivitis, a common complication in anophthalmic sockets^(17)^.

Most respondents recommended replacing EOPs every 1–5 years, particularly when edge damage was present. Proper prosthesis care, including annual cleaning and polishing, can extend the lifespan of an EOP by preserving surface smoothness and wettability, thereby minimizing conjunctival irritation when blinking^(17^,^19)^. Cleaning can be performed manually using water or mild surfactants, followed by air drying. The use of tissues or towels is discouraged due to the risk of surface scratches and microbial contamination^(1^,^3^,^31^,^32)^.

Web-based surveys offer a practical means of collecting data from a larger number of geographically dispersed participants^(6)^. However, this study had some limitations. Most notable among these was its reliance on self-reported practices from surgeons who responded voluntarily. Self-reported questionnaire responses can be less reliable than more objective means of data collection, while samples comprised only of respondents who chose to participate may not be generalizable to all practitioners in the given country. Interpretations should be based on the most commonly reported practices. However, this may inadvertently suggest that less frequently used approaches are inferior, which may not necessarily be the case.

Nonetheless, given the lack of standardized guidelines for the clinical management of anophthalmic sockets, this study provides important insights. The perspectives of Brazilian and Spanish oculoplastic surgeons regarding implant selection, clinical challenges, and EOP care may serve as a valuable reference for other practitioners, enhancing patient outcomes in this unique population.

In conclusion, while oculoplastic surgeons in Brazil and Spain largely employ comparable approaches to the management of anophthalmic sockets, differences were found in the types of implants used, the recommended frequency of prosthesis cleaning, and the recommendations given regarding the removal of EOPs before sleeping.

## Data Availability

The contents underlying the research text are included in the manuscript
